# Evolution of solidification texture during additive manufacturing

**DOI:** 10.1038/srep16446

**Published:** 2015-11-10

**Authors:** H. L. Wei, J. Mazumder, T. DebRoy

**Affiliations:** 1 Department of Materials Science and Engineering, The Pennsylvania State University, University Park, PA 16802, USA; 2 Center for Laser Aided Intelligent Manufacturing, University of Michigan, Ann Arbor, MI 48109, USA

## Abstract

Striking differences in the solidification textures of a nickel based alloy owing to changes in laser scanning pattern during additive manufacturing are examined based on theory and experimental data. Understanding and controlling texture are important because it affects mechanical and chemical properties. Solidification texture depends on the local heat flow directions and competitive grain growth in one of the six <100> preferred growth directions in face centered cubic alloys. Therefore, the heat flow directions are examined for various laser beam scanning patterns based on numerical modeling of heat transfer and fluid flow in three dimensions. Here we show that numerical modeling can not only provide a deeper understanding of the solidification growth patterns during the additive manufacturing, it also serves as a basis for customizing solidification textures which are important for properties and performance of components.

Additive manufacturing (AM), also known as 3D printing, is a promising technology for rapid production of net-shaped or near-net-shaped components from powders or wires melted by a high power density heat source. During laser assisted AM process, the metal powders are melted by a focused laser beam. The laser scanning pattern is controlled by a computer and the molten metal solidifies to form successive layers of deposition^1^. Current applications of AM include printing of mechanical components^2^, electronics^3^, tissues^4^,^5^, implants and prosthesis^6^. A wide range of materials have been used in AM including nickel based super alloys^7^, titanium alloys^8^,^9^, stainless steels^10^,^11^, and polymeric^12^ materials. Among these materials, nickel based alloys are extensively used in turbine blades, combustion chambers and nuclear reactors owing to their excellent tensile and creep properties as well as resistance to hot corrosion and oxidation^13^,^14^. Since texture affects mechanical and chemical properties of the fabricated components, its control is crucial for obtaining target properties in AM parts that cannot be made by any other means.

Components having the exactly the same shape and size can be made by different laser scanning patterns. However, since the traverse path of the laser beam affects solidification pattern, components of the exact same geometry can have strikingly different solidification patterns^15^,^16^. Dinda *et al.*^16^ reported that the solidification morphology of Inconel 718 was significantly affected by laser scanning patterns during deposition of a multiple layer laser component. The solidification pattern during unidirectional laser scanning resulted in the formation of primary dendrites at all layers at an angle 60° with the substrate. However, for bidirectional scanning, the growth direction of the primary dendrites altered in each layer by 90° with respect to the growth direction of primary dendrites in the previous layer^16^.

The solidification patterns depend on the local temperature field near the growth interface and the grain orientation of the substrate^17^,^18^. Previous work has shown that columnar grains grow epitaxially on single crystalline substrates^19^. Local temperature gradients and solidification growth rates at solid-liquid interfaces are significantly affected by the AM process parameters. High power density, low laser power and high scanning speed are beneficial for columnar grain growth epitaxial to the surface. The crystallographic orientation of the single crystal substrate also affects the orientation of the columnar dendrite^19^. In contrast, polycrystalline substrates contain many randomly oriented grains. For these substrates, the grain orientations are selected by competitive grain growth in one of the six <100> preferred growth directions parallel or nearly parallel to the local heat flow direction for cubic materials^20^,^21^.

A well-tested comprehensive heat transfer and fluid flow model can provide temperature fields, melt pool geometries and solidification parameters during AM processes. Here we show that numerical heat transfer and liquid metal flow calculations can provide an improved understanding of the evolution of solidification morphology and texture during multi-layer AM processes. By comparing the simulated heat flow directions with the corresponding solidification patterns of both unidirectional and bidirectional laser scanning processes, the mechanisms of formation of different solidification textures are examined.

The equations of conservation of mass, momentum and energy were solved^11^,^22^ in three-dimensions to calculate the temperature and velocity fields in laser assisted multiple layer AM. The model was used for both unidirectional and bidirectional laser scanning patterns. In the computational domain, the substrate was 150 mm long, 100 mm wide and 10 mm thick. The length and height of each layer were 50 mm and 0.2 mm, respectively. The computational domain of each layer was divided into 910 grid points in the scanning direction (x), 45 in the transverse direction (y) and 30 in the thickness (z) direction. The local values of the variables of each computational cell are related to the variable values of the neighboring cells with algebraic equations. The data used for the calculations is given in Table 1.

The direction of heat flow at any point on the solidification surface is normal to the surface. The predominant direction of heat flow is given by:


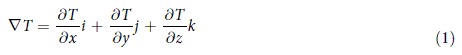


where *T* is temperature and *i*, *j* and *k* are unit vectors in the scanning (x), width (y) and vertical (z) directions, respectively. The temperature gradient, G, is:


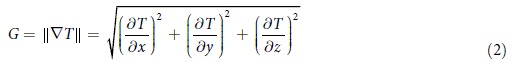


which is the magnititude of 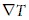. Note that there is no temperature gradient along the y-direction at any point on the longitudinal mid-section symmetry plane at y = 0. The solidification rate, R, at any location of the solid-liquid interface is:


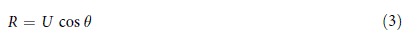


More details about the computation of heat transfer directions, solidification parameters, and heat transfer and fluid flow during AM are shown in the supplementary material. The angle, *θ*, between the heat flow direction and the horizontal line on this plane can be calculated from the following relation:


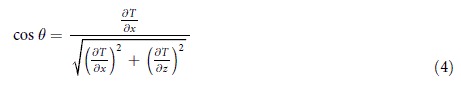


The normal solidification velocity 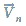 at the solid-liquid interface is geometrically related to laser scanning speed 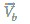
19:


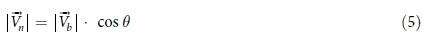


where *θ* is also the angle between the scanning and solidification directions. The relationship between the growth velocity *V*_*hkl*_ of the dendrite tip along a specific crystallographic direction [*hkl*] and the normal solidification velocity *V*_*n*_ is given by^19^:


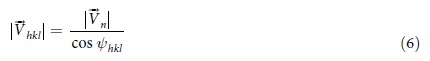


where *ψ*_*hkl*_ is the angle between the normal to the solidification front and the preferred [*hkl*] crystallographic direction.

The solidification morphology of nickel based alloy is dependent on G/R which is the ratio of the temperature gradient G and the solidification rate R at the solid-liquid interface. The solidification structures can be planar, cellular, columnar dendritic or equiaxed dendritic, depending on the cooling rate and the G/R values^20^. Values of G and R can be computed by equations (2) and (3) from the heat transfer and fluid flow model of AM. The calculated values of G/R at mid-length and mid-height along the longitudinal mid-section of all layers are in the range of 20 K s mm^−2^ to 100 K s mm^−2^. The minimum G/R value necessary for planar solidification is on the order of 7000 K s mm^−2^, which is estimated with the typical values for alloying element diffusion in liquid metal^20^. Therefore, a plane solidification front is unstable and the solidification structure will be either cellular or dendritic during this AM cooling process, which is consistent with the experimental observations that dendrites dominate the solidification structures.

Figure 1(a) shows the calculated temperature fields in a longitudinal mid-section of Inconel 718 deposition by unidirectional left to right laser scanning in all layers. Melt pool boundaries at different laser scanning positions of each layer are shown to demonstrate the evolution of the deposited track. It is a 5 mm part of the entire deposition length, which centers at the longitudinal mid-point of the track. The solidus and liquidus temperatures are 1533 K and 1609 K, respectively. The cross section grows slightly in size from left to right and from lower to upper layers because of accumulation of heat. Figure 1(b) shows the heat flow directions indicated in yellow vectors for various locations calculated using equation (4). The main heat flow directions at the solidification front are at an angle of about 60° with the horizontal substrate surface. This direction is perpendicular to the trailing edge of the melt pool which is oriented at about 30°angle with the horizontal line.

Figure 1(c,d) show the calculated solidification pattern of primary dendrites and the optical micrograph of the as-deposited Inconel 718 sample, respectively. The optical micrograph shows that the growth direction of the primary dendrites was at an angle of about 60° with the horizontal line, which coincides with the maximum heat flow direction at the solidification front. Note that the grains were randomly oriented in the polycrystalline substrate. During the solidification process of the first layer, grains may grow with a range of orientations at the solid-liquid interface. The optimal grain orientation is given by the minimum value of *ψ*_*hkl*_ which is 0. However, grains with other orientations, i.e., *ψ*_*hkl*_ greater than 0, can also grow, although to a lesser extent. A selection process occurs during the initial several layers, where the maximum heat flow direction is at about 60° with the horizontal plane in all the deposited layers. The primary dendrites obtain competitive growth along one of the six <100> preferred growth directions that is closely aligned to the maximum heat flow direction. Note that near the top surface, the solidification direction is nearly horizontal, i.e., almost parallel to the laser scanning direction. However, the top part of deposit is remelted when a layer is deposited above. This region is not preserved in the deposited part except at the topmost layer.

Figure 2(a) shows the calculated temperature fields in a longitudinal mid-section during bidirectional laser scanning for the deposition of Inconel 718. In each successive layer, the cross sections are similar in geometry but oriented in different directions because of the alternate scanning directions. The length and position of the deposit shown is similar to that of the unidirectional scanning. Figure 2(b) shows the calculated temperature and velocity fields of a melt pool at the mid-length the first layer, where laser beam moves from the left to the right. Figure 2(c) shows the calculated temperature and velocity fields of a melt pool at the mid-length of the second layer, which is formed by laser scanning from right to left. As shown in both Fig. 2(b,c), the liquid metal flows from the middle to the periphery at the melt surface driven by Marangoni effect resulting from the spatial gradient of surface tension due to local temperature variation on the melt pool surface. The velocities are of the order of 150 mm/s and at these velocities most of the heat is carried by convection which is the primary mechanism of heat transfer within the melt pool. Also, the melt pool is well mixed due to strong Marangoni convection. Melt pool in Fig. 2(c) is similar to that in Fig. 2(b) except that its orientation is different because of the reversal of the scanning direction with respect to the previous layer. The trailing edge of the melt pool in Fig. 2(c) has an angle of about −30° with the horizontal line, which is opposite to the angle in Fig. 2(b).

During bidirectional laser scanning, primary dendrite growth pattern is somewhat different from unidirectional scanning. In the first layer, the nucleation and primary dendrite growth mechanism for bidirectional laser scanning is the same as that of unidirectional laser scanning. However, additional primary dendrite growth patterns are of interest in the subsequent layers. Figure 3(a) shows the calculated directions of maximum heat flow for alternate laser scanning directions. Grains whose easy-growth directions align closely with the maximum heat flow direction at the solid-liquid interface achieve competitive growth during the solidification process. As can be seen from Fig. 3(a), the angles for maximum heat flow direction with horizontal line are about 60° for left to right laser scanning directions, and −60° for right to left laser scanning directions. Thus, it is worth examining if the primary dendrite growth directions in neighboring layers will follow these heat flow directions and form the corresponding solidification textures.

Figure 3(b) schematically shows different primary dendrite growth patterns. Solidification pattern 1 shows primary dendrite trunks coinciding with directions of maximum heat flow in different layers. For each layer the directions of dendrite growth are influenced by the direction of scanning. If solidification were to occur according to pattern 1, the angle between primary dendrite trunks of neighboring layers should be 120° which is inconsistent with the experimental observations. Solidification pattern 1 does not occur because the driving force required for nucleation is much higher than that for grain growth. In solidification pattern 2, the primary dendrites of neighboring layers grow perpendicular to each other. The orientation of grains in the first layer is 60° with horizontal line which is consistent with the maximum heat flow direction. The initiation of primary dendrites in the second layer takes advantage of the secondary dendrites of the first layer and grows epitaxially on them. Primary and secondary dendrites are perpendicular to each other and both are oriented in easy-growth directions. Note that the primary dendrite growth direction deviates 30° from the maximum heat flow direction in the second layer in solidification pattern 2. Solidification pattern 2 does not form because of large misalignment (30^o^) with the maximum heat flow direction for it to be a viable option.

Solidification pattern 3 shows a somewhat similar condition to solidification pattern 2, except the grain orientation in the first layer which is at 45° with horizontal line. Because the primary dendrites are at an angle of 45° with the horizontal in the first layer, the primary dendrite which grows perpendicular to it in the second layer makes an angle of −45° with the horizontal line. As a result, there is a deviation of 15° between the primary dendrite growth direction and the maximum heat flow direction in all the layers. The resulting solidification texture is shown in Fig. 4(a) and the corresponding optical micrograph is shown in Fig. 4(b). It shows that the primary dendrite orientations are slightly misaligned with the maximum heat flow directions but the solidification texture is consistent with solidification pattern 3 in Fig. 3(b). Here, the primary dendrites grow at 45° angle with the horizontal direction in the first layer. In the second layer, the primary dendrites are aligned with the secondary dendrites of the first layer, i.e., with −45° orientation with the horizontal direction. Thus, in pattern 3, there is a deviation of 15° from the maximum heat flow direction in all layers and this pattern is selected in practice.

In summary, the solidification textures of a nickel based alloy during additive manufacturing could be controlled using numerical modeling of heat transfer and liquid metal flow. Since texture affects mechanical and chemical properties, its control based on scientific principles is important for serviceability of the fabricated parts. Primary dendrite orientation of about 60° with the horizontal plane was calculated from the numerical model for unidirectional laser scanning for all depositing layers. During bidirectional laser scanning, the angle between primary dendrites of neighboring layers was about 90°. The numerical modeling results help to understand the mechanism of formation of the solidification texture during unidirectional and bidirectional laser scanning patterns and provide a basis for customizing solidification textures during additive manufacturing.

## Additional Information

**How to cite this article**: Wei, H. L. *et al.* Evolution of solidification texture during additive manufacturing. *Sci. Rep.* **5**, 16446; doi: 10.1038/srep16446 (2015).

## Supplementary Material

Supplementary Information

## Figures and Tables

**Figure 1:**
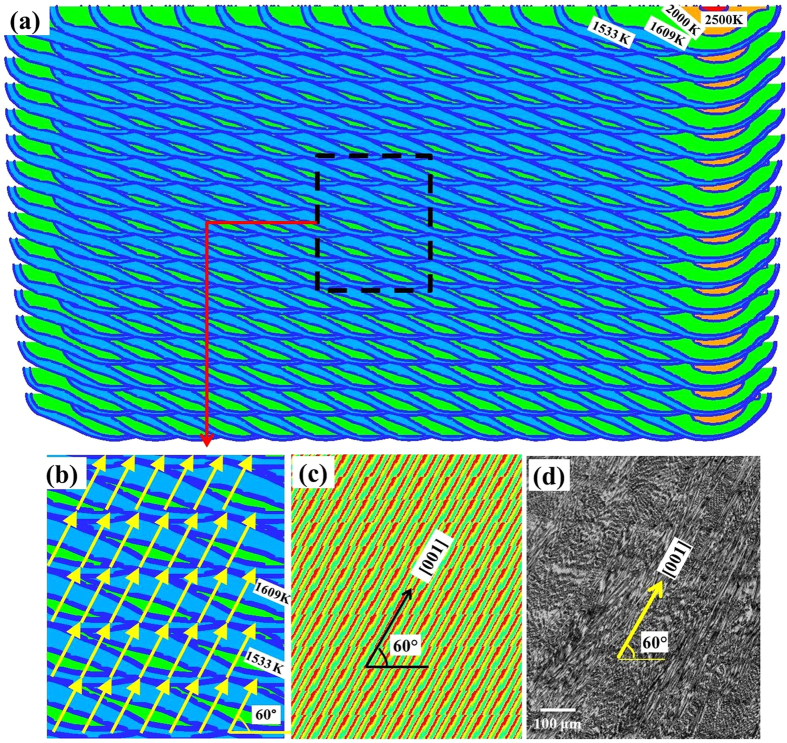
Maximum heat flow directions and solidification textures for unidirectional laser scanning. (**a**) Calculated longitudinal sections at various locations for multiple layer single track Inconel 718 deposition. (**b**) Magnification of calculated temperature field with maximum heat flow directions indicated in yellow vectors. (**c**) Calculated solidification pattern of primary dendrites. (**d**) Optical micrograph of the as-deposited Inconel 718 sample.

**Figure 2:**
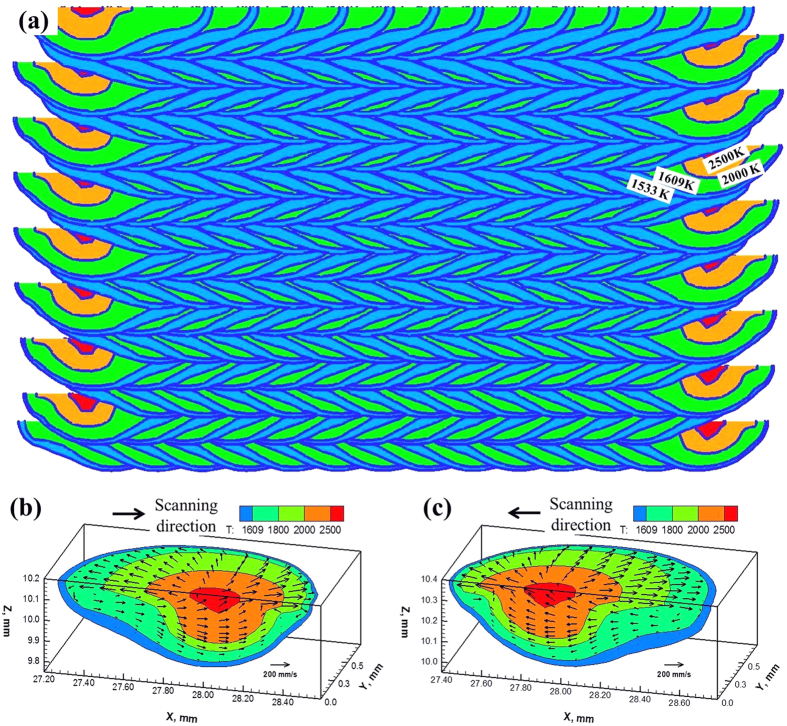
Temperature and velocity fields for bidirectional laser scanning. (**a**) Calculated longitudinal sections at various locations for multiple layer single track Inconel 718 deposition. (**b**) Three dimensional melt pool at the mid-length of the first layer. (**c**) Three dimensional melt pool at the mid-length of the second layer.

**Figure 3:**
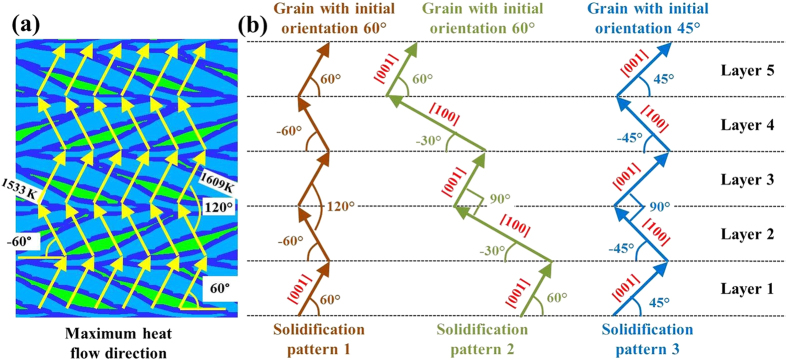
Maximum heat flow directions and solidification patterns for bidirectional laser scanning. (**a**) Calculated temperature field with maximum heat flow directions indicated in yellow vectors. (**b**) Schematic illustration for primary dendrite growth patterns of grains with different orientations during bidirectional laser scanning.

**Figure 4:**
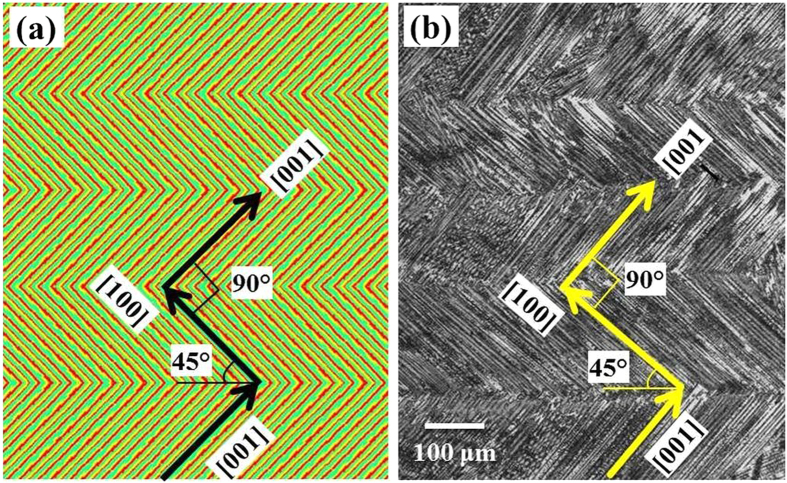
Solidification textures for unidirectional laser scanning. (**a**) Calculated solidification patterns of primary dendrites. (**b**) Optical micrograph of the as-deposited Inconel 718 sample for bidirectional laser scanning.

**Table 1:** Materials properties used for numerical calculations.

Parameters	Value
Laser power (W)	750
Laser beam diameter (mm)	0.5
Laser distribution factor	3
laser scanning speed (mm s^−1^)	6.25
Liquidus temperature (K)	1533
Solidus temperature (K)	1609
Density of metal (kg m^−3^)	8100
Thermal conductivity of solid (J m^−1^ s^−1^ K^−1^)	0.5603 + 0.0294*T*-7.0×10^−6^*T*^2^
Thermal conductivity of liquid (J m^−1^ s^−1^ K^−1^)	29.6
Specific heat of solid (J kg^−1^ K^−1^)	360.56 + 0.2621*T*-4×10^−5^*T*^2^
Specific heat of liquid (J kg^−1^ K^−1^)	720.0
Temperature coefficient of surface tension (N m^−1^ K^−1^)	−0.37×10^−3^
Coefficient of thermal expansion (K^−1^)	1.63×10^−5^
Viscosity of liquid (kg ms^−1^)	7.0×10^−3^

## References

[b1] Hofmann D. C. *et al.* Developing gradient metal alloys through radial deposition additive manufacturing. Sci. Rep. 4, 5357 (2014). 24942329 10.1038/srep05357 PMC4062900

[b2] Hofmann D. C. *et al.* Compositionally graded metals: A new frontier of additive manufacturing. J. Mater. Res. 29, 1899–1910 (2014).

[b3] Zheng Y., He Z. Z., Gao Y. X. & Liu J. Direct Desktop Printed-Circuits-on- Paper Flexible Electronics. Sci. Rep. 3, 1786 (2013).

[b4] Murphy S. V. & Atala A. 3D bioprinting of tissues and organs. Nat. Biotechnol. 32, 773–785 (2014). 25093879 10.1038/nbt.2958

[b5] Miller J. S. *et al.* Rapid casting of patterned vascular networks for perfusable engineered three-dimensional tissues. Nat. Mater. 11, 768–774 (2012). 22751181 10.1038/nmat3357 PMC3586565

[b6] He Y., Xue G. H. & Fu J. Z. Fabrication of low cost soft tissue prostheses with the desktop 3D printer. Sci. Rep. 4, 6973 (2014). 25427880 10.1038/srep06973 PMC4245596

[b7] Liu Z. & Qi H. Numerical Simulation of Transport Phenomena for a Double-Layer Laser Powder Deposition of Single-Crystal Superalloy. Metall. Mater. Trans. A. 45, 1903–1915 (2014).

[b8] Xu W. *et al.* Additive manufacturing of strong and ductile Ti–6Al–4V by selective laser melting via *in situ* martensite decomposition. Acta Mater. 85, 74–84 (2015).

[b9] Carroll B. E., Palmer T. A. & Beese A. M. Anisotropic tensile behavior of Ti–6Al–4V components fabricated with directed energy deposition additive manufacturing. Acta Mater. 87, 309–320 (2015).

[b10] Yan C., Hao L., Hussein A., Young P. & Raymont D. Advanced lightweight 316L stainless steel cellular lattice structures fabricated via selective laser melting. Mater. Design. 55, 533–541 (2014).

[b11] Manvatkar V., De A. & DebRoy T. Spatial variation of melt pool geometry, peak temperature and solidification parameters during laser assisted additive manufacturing process. Mater. Sci. Tech. 31, 924–930 (2015).

[b12] Melchels F. P. W. *et al.* Additive manufacturing of tissues and organs. Prog. Polym. Sci. 37, 1079–1104 (2012).

[b13] Jia Q. & Gu D. Selective laser melting additive manufacturing of Inconel 718 superalloy parts: Densification, microstructure and properties. J. Alloy. Comp. 585, 713–721 (2014).

[b14] Zhang Y. N., Cao X., Wanjara P. & Medraj M. Oxide films in laser additive manufactured Inconel 718. Acta Mater. 61, 6562–6576 (2013).

[b15] Dinda G. P., Dasgupta A. K. & Mazumder J. Laser aided direct metal deposition of Inconel 625 superalloy: Microstructural evolution and thermal stability. Materials Science and Engineering: A. 509, 98–104 (2009).

[b16] Dinda G. P., Dasgupta A. K. & Mazumder J. Texture control during laser deposition of nickel-based superalloy. Scripta Mater. 67, 503–506 (2012).

[b17] David S. A. & Debroy T. Current issues and problems in welding science. Science. 257, 497–502 (1992). 17778680 10.1126/science.257.5069.497

[b18] Debroy T. & David S. A. Physical processes in fusion welding. Rev. Mod. Phys. 67, 85–112 (1995).

[b19] Liu Z. & Qi H. Effects of substrate crystallographic orientations on crystal growth and microstructure formation in laser powder deposition of nickel-based superalloy. Acta Mater. 87, 248–258 (2015).

[b20] Kou S. Welding metallurgy 2nd edn. (John Wiley & Sons, New Jersey, 2003).

[b21] Blecher J. J., Palmer T. A. & DebRoy T. Solidification Map of a Nickel-Base Alloy. Metall. Mater. Trans. A. 45, 2142–2151 (2013).

[b22] Manvatkar V., De A. & DebRoy T. Heat transfer and material flow during laser assisted multi-layer additive manufacturing. J. Appl. Phys. 116, 124905 (2014).

