# Supplementary material for: Evolution of solidification texture during additive manufacturing
Source: Sci Rep. 2015 Nov 10;5:16446. doi: 10.1038/srep16446 (PMC4639736; doi:10.1038/srep16446)
Supplement: Supplementary Information [file srep16446-s1.pdf]

Supplementary information on the evolution of solidification texture during additive manufacturing

H. L. Wei [1], J. Mazumder[2] & T. DebRoy[1,*]

[1]Department of Materials Science and Engineering, The Pennsylvania State University, 309 Forest Resources Lab, University Park, PA 16802, USA
[2]Center for Laser Aided Intelligent Manufacturing, University of Michigan, Ann Arbor, MI 48109, USA

[*] Corresponding author: debroy@psu.edu

Supplementary Information includes:

Supplementary Discussion 1-3

Supplementary Equations 1-3

Supplementary Figure 1-2

Supplementary References

Solidification structures of nickel based alloys during additive manufacturing are significantly affected by the directions of heat flow and solidification parameters such as temperature gradient and solidification rate at the solid liquid interfaces. Direct measurement of maximum heat flow directions and solidification parameters are difficult due to the movement of the melt pool and the interactions between the powder and the laser beam. A recourse is to use a well-tested comprehensive heat transfer and fluid flow model to calculate temperature fields and melt pool geometries during AM processes for various depositing materials[1,2]. The maximum heat flow direction, temperature gradient and the solidification rate can be calculated based on the transient, three-dimensional heat transfer and liquid metal flow calculations.

Supplementary Discussion 1. Heat transfer and fluid flow model of AM

A. Model assumptions

Several simplifying assumptions are made to make the complex, three-dimensional, transient calculations tractable. The densities of the solid and liquid metals are assumed to be constant. The surface of the growing layer is assumed to be flat. The loss of alloying elements due to vaporization and its effects on both the heat loss and composition change are not considered in the calculations.

B. Governing equations

The model solves the conservation equations for mass, momentum, and energy in transient three-dimensional form. These equations are available in standard text books[3] and in many of our previous publications[4,5]. The specific discretization scheme and the solution methodology for transient three dimensional form are also discussed in details in the literature[3,4]. Spatially non-uniform grids, with finer grid spacing near the axis of the laser beam were used for efficient calculation of variables. The governing equations were discretized by following a control volume method[3]. The velocity components and the scalar variables were stored at different locations to enhance the convergence and stability of the computational scheme. At each time step, the three components of velocities and the enthalpy were iterated following a sequence known as the SIMPLE algorithm[3]. The implicit computational scheme adapted is unconditionally stable. The discretized linear equations were solved using a Gaussian elimination technique known as the tri-diagonal matrix algorithm[3].

C. Computational domain and calculation procedure

The transient heat transfer and fluid flow calculations are performed for a rectangular solution domain representing the substrate, deposited layers, and the surrounding gas shown in Supplementary Fig. 1. The deposition process is simulated through discrete time steps. The moving heat source is simulated by progressively shifting of the laser beam axis by a very short predetermined distance, X_s, in the direction of deposition equal to a small fraction of the laser beam diameter. The laser beam shifting direction and X_s value are same for all the layers during unidirectional laser scanning. During bidirectional laser scanning, the shifting directions of the laser beam are opposite for neighboring layers and the value of X_s is identical for all the layers. The corresponding time step, Δt, is calculated from the scanning velocity, v:

$$\Delta t = X_s / v \quad (1)$$

During each shift, the properties of the computational cells representing the volume of the deposited material are changed from the properties of the gas to that of the deposit material. At the end of each layer, an idle time is provided to allow the laser beam to move to the initial location prior to the deposition of the next layer. The aforementioned procedure is repeated till the deposition of all the layers.

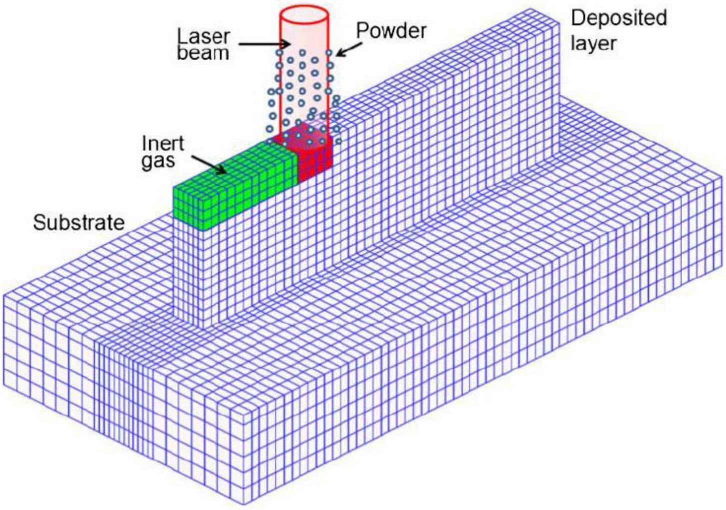

Supplementary Figure 1. A schematic representation of the solution domain[2].

D. Boundary conditions and convergence criteria

At the beginning of the simulation, all the cells above the substrate are assigned properties of an inert gas and the initial temperature of the domain is taken as the room temperature (298 K). The variation of all variables across the mid-section longitudinal symmetry plane is set to zero. In the remaining surfaces, heat loss by radiation and convection is applied as boundary conditions for the solution of the enthalpy equation. At the top surface of the melt pool, the velocities arising from the surface tension variation due to temperature gradient are applied for the solution of momentum equations[5,6]. Velocities are set to be zero at other surfaces since they are solid and the melt pool does not extend there.

At any given time step, the iterations were terminated when two convergence criteria were satisfied. The magnitudes of the residuals of enthalpy and the three components of velocities, and the overall heat balance were checked after every iteration. The largest imbalance of any variable on the two sides of a discretization equation for all interior grid points had to be less than 0.1%. In addition, the overall

heat balance criterion required that the sum of the total heat loss from the domain and the heat accumulation had to be almost equal to the heat input into the calculation domain. Their difference had to be less than 0.5% of the heat input for this convergence criterion to be satisfied. The criteria were selected so that the final results were not adversely affected while maintaining computational speed.

Supplementary Discussion 2. Calculation of maximum heat flow directions during AM process.

Directions of heat transfer at the solidification interface can be calculated based on the temperature fields from the heat transfer and fluid flow model. Supplementary Fig. 2 shows a schematic representation of a melt pool with the angle θ between the laser scanning direction and the maximum heat flow direction, as well as the angle ψ_{hkl} between the normal to the solid-liquid interface and the preferred [*hkl*] crystallographic direction for dendrite growth.

The direction of heat flow at any point on the solidification surface is normal to the surface. The predominant direction of heat flow is given by:

$$\nabla T = \frac{\partial T}{\partial x} i + \frac{\partial T}{\partial y} j + \frac{\partial T}{\partial z} k \tag{2}$$

where T is temperature and i, j and k are unit vectors in the scanning (x), width (y) and vertical (z) directions, respectively. The angle, θ, between the heat flow direction and the horizontal line at any point on the solidification surface can be calculated from the following relation:

$$\cos\theta = \frac{\frac{\partial T}{\partial x}}{\sqrt{\left(\frac{\partial T}{\partial x}\right)^2 + \left(\frac{\partial T}{\partial y}\right)^2 + \left(\frac{\partial T}{\partial z}\right)^2}} \quad (3)$$

Therefore, the maximum heat flow directions at the solid-liquid interface for any location can be calculated based on the three dimensional temperature fields. The temperature gradient is 0 along the y direction at any point on the longitudinal mid-section as it is the symmetric plane for the melt pool along the melt pool width direction. Thus, the calculation of the maximum heat flow directions becomes a two dimensional issue for this particular plane and the results are demonstrated in the main article.

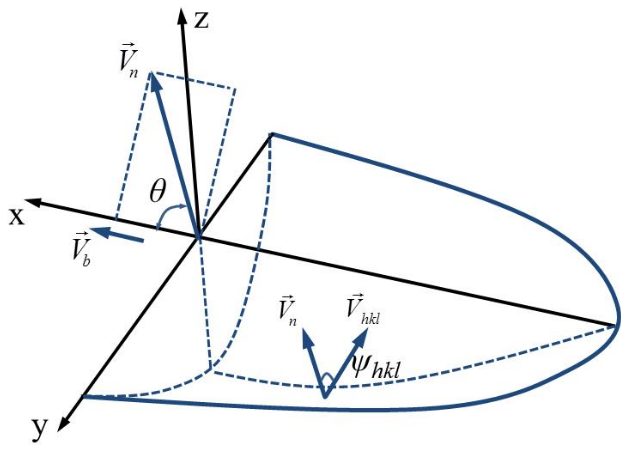

Supplementary Figure 2. Schematic representation of a melt pool with the angle θ between the laser scanning direction and the maximum heat flow direction which is also normal solidification velocity $\vec{V}_n$ here, as well as the angle ψ_{hkl} between the normal to the solid-liquid interface and the preferred [*hkl*] crystallographic direction for dendrite growth.

Supplementary Discussion 3. Calculation of solidification parameters during AM process.

Solidification morphologies and solidification structure dimensions are dependent on temperature gradient G and solidification rate R at the solid-liquid interface. GR determines the size of the solidification structures and G/R determines the solidification mode, i.e. planar, cellular, columnar dendritic and equiaxed dendritic.

During rapid solidification of the melt pool for AM, the undercooling of liquid metal is a critical parameter that controls the solidification microstructure and segregation effects. The melt pool solidification during takes places from the preexisting substrate or pre-deposited layer. The undercooling associated with solidification results from thermal, constitutional, kinetic and solid curvature effects. An accurate modeling of the AM melt pool solidification requires coupling of a solidification model with the heat transfer and fluid flow model. In the present study, the equilibrium liquidus isotherm is assumed to represent the liquid/mush zone boundary, while the equilibrium solidus isotherm corresponds to the mush zone/solid boundary. The solidification parameters presented in this work were calculated by considering the heat transfer and fluid flow within the melt pool during AM process.

Supplementary References:

1. Manvatkar, V., De, A. & DebRoy, T. Heat transfer and material flow during laser assisted multi-layer additive manufacturing. *J. Appl. Phys.* **116,** 124905 (2014)

2. Manvatkar, V., De, A. & DebRoy, T. Spatial variation of melt pool geometry, peak temperature and solidification parameters during laser assisted additive manufacturing

process. *Mater. Sci. Tech.* **31,** 924-930 (2015)

3. Patankar, S. V. *Numerical heat transfer and fluid flow*. (McGraw-Hill, New York, 1982)

4. Zhang, W., Kim, C.-H. & DebRoy, T. Heat and fluid flow in complex joints during gas metal arc welding—Part I: Numerical model of fillet welding. *J. Appl. Phys.* **95,** 5210-5219 (2004)

5. Zhang, W., Roy, G. G., Elmer, J. W. & DebRoy, T. Modeling of heat transfer and fluid flow during gas tungsten arc spot welding of low carbon steel. *J. Appl. Phys.* **93,** 3022-3033 (2003)

6. Raghavan, A., Wei, H. L., Palmer, T. A. & DebRoy, T. Heat transfer and fluid flow in additive manufacturing. *Journal of Laser Applications*. **25,** 052006 (2013)

7. Kou, S. *Welding metallurgy 2nd edn*. (John Wiley & Sons, New Jersey, 2003)
